# Navigating Society 5.0: Integrating Strength-Based Leadership and Job Crafting for Improved Nursing Outcomes

**DOI:** 10.1155/jonm/4986260

**Published:** 2025-08-05

**Authors:** Xule Wang, Wei Liu

**Affiliations:** ^1^Medical College, Xuchang University, Xuchang 461000, China; ^2^Yunnan Agricultural University, Kunming 650201, China

**Keywords:** artificial intelligence, creativity, job crafting, Society 5.0, strength-based leadership

## Abstract

**Aim:** This study investigates the impact of strength-based leadership (SBL) on fostering AI-driven job crafting to enhance patient care and nurse creativity in the context of Society 5.0.

**Background:** The emergence of Society 5.0 emphasises a human-centred approach to technological integration, posing opportunities and challenges for nursing management. The increasing reliance on digital technologies necessitates that nurses update their skills and adapt to new systems, potentially compromising the quality of care and personal connections with patients.

**Methods:** A cross-sectional descriptive study design was employed to target nurses in tertiary hospitals in Henan province, China. The sample size was determined using G∗power software, accounting for a projected dropout rate. The structural equation modelling approach was adopted to analyse the collected data.

**Results:** The results show that SBL has positively promoted AI-driven crafting (task, relational and cognitive). Furthermore, the outcomes highlight that AI-driven job crafting positively promotes patients' quality of care. Surprisingly, the findings indicate that AI-driven job crafting (task and cognitive) has an insignificant relationship with nursing creativity.

**Conclusion:** By elucidating the role of SBL in promoting AI-driven job crafting, this research aims to provide insights into effective nursing management strategies that leverage technological advancements while maintaining high standards of patient care.

**Implications for Nursing Management:** The findings highlight the importance of leadership focussing on employee strengths. This fosters an environment conducive to innovation and improved patient outcomes in a rapidly evolving healthcare landscape.

## 1. Introduction

Over the centuries, humanity has significantly progressed in science and technological development. The ongoing evolution has been a critical factor for human well-being in various societal revolutions, culminating with the birth of Society 5.0 (S5.0). The Japanese government introduced the S5.0 concept, which envisions a human-centred society in which technological breakthroughs are interwoven into daily life to improve well-being and alleviate social issues [1]. Notwithstanding a growing interest in the societal effects of digital technologies, the scientific literature contains gaps that have led to the emergence of the paradigm shift known as S5.0 [2, 3]. Amalia and Susanti [4] contended that S5.0 anticipates a future in which sophisticated technology amiably interacts with human existence to build a more intelligent, prosperous society. Fontes et al. [5] argued that S5.0 expands Society 4.0, which evolved with the advent of digital technologies with a profit-driven focus. Recently, the challenges related to nursing management have gained prominence in the S5.0 human-centric approach that integrates digitalisation to enhance well-being and patient care. Furthermore, S5.0 enables patients to manage their well-being through individualised information and constant monitoring [1]. Consequently, existing nursing strategies are insufficient to tackle the challenges in S5.0. Furthermore, the significance of S5.0 on the Chinese nursing profession highlights several challenges, including resistance to changing traditional nursing practices [6, 7]. China needs to address these gaps to fully reap the full advantages of S5.0 in healthcare.

In healthcare and technology, digitalisation has revolutionised nursing creativity and patient care [8]. In this context, the primary issue is that nurses need to update their abilities to adopt new systems. Such a continual shift can hinder their ability to think creatively about patient care. Furthermore, emphasising data entry and technological administration may diminish nurses' time with patients [9]. Consequently, this jeopardises the quality of care (QOC) and the personal connections required for practical nursing. Additionally, a more transactional approach to care may result from a dependence on digital technology, where interactions are more concerned with getting things done than building relationships [10, 11]. This change may compromise nursing's holistic and compassionate facets, making it more challenging for nurses to offer individualised care that considers each patient's particular requirements.

Several approaches have been brought forward to address the challenges of creativity and patient care. For instance, Liu and Tong [12] argued that the leader's role is critical to promote employee innovation and a growth mindset. Wang et al. [13] contented that leadership plays a significant role for employee retention. In the prior literature, various leadership behaviours do not explicitly indicate whether they are subordinate's strength-focused or subordinate's deficit-focused [13]. Thus, the recent literature suggests that strength-based leadership (SBL) emerges to address the leaders' and subordinates' behaviour. According to Ding et al. [14], SBL is a positive leadership approach that emphasises recognising, fostering and leveraging the strengths of leaders and their subordinates to improve subjective experiences and assist firms in gaining a sustained competitive advantage. In nursing, SBL helps Chinese nurses to mitigate the work fatigue and turnover intention [13]. Lavoie-Tremblay et al. [15] affirmed that SBL programs promote well-being and work satisfaction among nurses and healthcare leaders. In other domain, SBL promotes employee engagement [13], innovative behaviour [12] and knowledge sharing behaviour [16].

In the age of artificial intelligence (AI), SBL emerges as a motivational force that drives a firm's long-term success. Therefore, job crafting in the AI or AI crafting context has gained significant attention [17]. Dong et al. [18] define an individual's spontaneous actions to shape and redefine their job in response to AI. In other words, AI crafting enables people to share, evolve and redefine their jobs, reclaiming flexibility and finding more profound meaning in their work [19]. Furthermore, AI-driven job crafting is a proactive strategy, allowing employees to retain an immense sense of sovereignty over their responsibilities and duties [20]. In the prior literature, AI-driven job crafting has gained limited attention, especially in nursing. For instance, Dong et al. [18] claimed that AI drivers (talk, leader attention and self-efficacy) significantly enhance the AI crafting among Chinese employees. Cheng et al. [19] affirmed that firm AI adoption enhances promotion-focused job crafting. Handke et al. [21] claimed that information and communication technology is the key driving tool for job crafting. Li et al. [17] affirmed that leaders' AI crafting promotes employee AI crafting to enhance employee engagement and help. In the medical domain, Perez et al. [22] argued that AI alters work aspects, meaning that employees find in their work and redefines employee identity. Song et al. [23] argued that the higher interdependence between humans and robots enables employees to change their traditional work procedures. Prior job crafting literature suggests that employees are not reactive receivers of environmental changes but can be proactive crafters [17]. Therefore, to cope with AI changes, employees may proactively cope with job design boundaries and alternation brought by AI, which helps them gain control over their job [17]. However, there is scant evidence of AI crafting in the nursing profession. Given the highlighted knowledge gaps in the existing literature, this study aims to investigate the impact of SBL in fostering AI-driven job crafting or AI crafting to improve patient care and nurse creativity.

## 2. Theory and Hypotheses

### 2.1. Job Demands–Resources (JD-R)

The theoretical foundation of this study is firmly grounded on the JD-R model [24]. The fundamentals of JD-R suggest that working conditions can be divided into job demands and resources [25]. The notion of job demands refers to the emotional, physical, organisational or social aspects of a job that needs persistent physical and/or emotional effort or abilities and is thus associated with specific physiological and psychological costs [26, 27]. Whereas, job resources are a job's mental, social, physical or organisational aspects that aid in attaining work objectives, reducing job demands and their associated physiological and psychological costs or promoting personal growth, understanding and development [25]. According to Kessler [27], many people are motivated to protect and acquire resources because they meet their basic psychological requirements for independence, relatedness and competency. Furthermore, job resources are divided into three levels: firm (career progression), interpersonal (supervisor and coworker support) and task (task identity and significance) [28]. Additionally, Kessler [27] stated that in the JD-R model, employees can actively change the content or framework of their jobs by preferring specific activities, negotiating changing responsibilities or attaching personal meaning to their task or position. The process in which employees actively shape their work is known as job crafting [26–29].

The JD-R model has been well grounded in addressing the unique challenges nurses face in nursing. For instance, Wang et al. [13] explained the role of SBL to minimise nurse's turnover intention through motivation and health impairment processes. Zhang et al. [9] highlight the association between job crafting and work engagement among Chinese nurses through the JD-R model. Similarly, Iida et al. [28] examined the linkages between team job crafting and work engagement among Japanese nurses under the lens of the JD-R model. Through the JD-R model, Lee and Hwang [30] examined the factors influencing patient safety management among nursing care in the COVID-19 pandemic. Finally, Johari et al. [31] explained the relationship between job characteristics, work engagement and emotional intelligence among Malaysian nurses through the JD-R model. Conclusively, this study uses the JD-R model to explain why SBL, as a vital job resource, may influence nurses' creativity and patient care via the mediating mechanisms of AI-driven job crafting.

### 2.2. Job Crafting in AI or AI Crafting

With the rising prevalence of AI, scholars are growing interested in how AI's application transforms the work design known as AI crafting. According to Dong et al. [18], AI crafting represents the individual's proactive behaviour in changing their work boundaries and practices related to AI. Li et al. [17] define AI crafting as an individual deliberate act to shape, mould and redefine a job in response to AI. Based on the above definitions, AI crafting represents a proactive behaviour in that employees are not passive receivers of environmental changes but can be proactive crafters [22]. For instance, service robots in the hospitality industry can process and serve customers' orders. Therefore, employees can proactively change their jobs and focus on other aspects [17]. Cheng et al. [19] argued that applying AI transforms the firm environment from static to dynamic. Consequently, employees face a dynamic work environment full of uncertainty and complexity [21]. Employees may proactively modify their jobs and tasks [19]. Moreover, the human-centric aspect of Industry 5.0 stresses the interdependence between humans and technology [32]. In a highly interdependent working environment, individuals are more willing to change their traditional work procedures [23]. Perez et al. [22] affirmed that AI crafting is an opportunity to reposition and reclaim the meaning of the profession.

From the JD-R perspective, the workplace application of AI can alter job resources (flexibility and autonomy) as well as job demands (workload) [21]. A relevant example is ‘zoom fatigue', which is that the frequent utilisation of videoconferencing can increase job demands because of the additional focus required to interpret meaning without full contextual clues. Moreover, Perez et al. [22] affirmed that AI may offer the potential to minimise the routine tasks (job demand) and benefit employee productivity and performance (job resources). However, this perspective remains relatively unexplored on how AI crafting enhances employee and patient outcomes, especially in nursing. Finally, Table 1 shows a literature review on the relationship between AI and job crafting.

### 2.3. SBL and Job Crafting (AI Crafting)

The notion of SBL has attracted much attention lately as positive psychology has moved forward. The concept of SBL refers to the “*degree to which leaders deliberately promote followers' strength identification, development and deployment*” [14, 41]. Fundamentally, the SBL has three tenants: (1) leaders ought to commit their time and attention to developing their followers' strengths; (2) they must form varied teams with expertise in execution, persuasion, connection development and strategic thinking and (3) they must grasp the value of establishing confidence, believe and enthusiasm in their followers [41, 42]. SBL distinguishes itself from other leadership styles, such as leader–member exchange (LMX), authentic leadership, humble leadership and transformational leadership, by emphasising strengths' improvement and equitable relationships with subordinates [43]. Nevertheless, while these leadership styles acknowledge and promote abilities, they fail to emphasise the leader's active commitment to developing and exploiting oneself and their fellow employees' abilities [16]. The extent of the literature highlights that SBL promotes employee resilience [43], well-being [41], career satisfaction [41] and knowledge sharing [16]. However, there is a lack of research on the association between SBL and job crafting.

Prior research describes AI crafting as employees' actions to change their work and task relationships to improve their mental awareness of the job purpose and achieve a better person-job fit [44]. Park and Park [45] claimed that AI crafting is a form of proactive work behaviour in which people actively change their job-related traits. Generally, job crafting has three forms: task, relational and cognitive [45]. Task crafting (TC) refers to the individual behaviour to actively change the type, number and scope of the work [13], whereas relational crafting (RC) refers to alter the ways of interacting with others at workplace [13]. Finally, cognitive crafting (CC) refers to change attitudes towards current work tasks to perceive work as meaningful [13]. Iida et al. [28] argued that these three forms are linked with individual job crafting. In the nursing domain, job crafting has an association with burnout [44], engagement [9, 28], job embeddedness [46] and commitment [47]. Moreover, Alwali [48] contended that the servant leadership style positively promotes employee job crafting. Likewise, Alwali [49] affirmed that transformational leadership positively moderates the relationship between psychological capital and innovative work behaviour. However, there is a lack of research on the relationship between job crafting and SBL. To fill this void, the following hypotheses have been proposed.

  H1a: SBL has a positive association with TC.
  H1b: SBL has a positive association with RC.
  H1c: SBL has a positive association with CC.

### 2.4. Job Crafting, Patients' QOC and Employee Creativity

Prior literature indicated that job crafting is a beneficial construct at the firm and individual levels. As shown in the previous section, job crafting classified into task (individual behaviour to actively change the type, number and scope of the work), relational (alter the ways of interacting with others at workplace) and cognitive (to alter attitudes towards the current work tasks to perceive work as meaningful) [13]. Prior literature examined job crafting at the individual level. For instance, Guo et al. [44] examined the role of job crafting to minimise burnout among Chinese nurses. Similarly, Zhang et al. [9] examined the role of job crafting in promoting Chinese nurses' engagement. Likewise, Felder et al. [50] argued that job crafting is an employee retention strategy in nursing due to a lack of appreciation. Wu et al. [51] affirmed that job crafting promotes nurses' innovative behaviour during crises. At the firm level, job crafting helps organisations to gain a competitive advantage. For instance, Yun et al. [46] affirmed that job crafting promotes job embeddedness and social support in South Korea. Saleh et al. [47] affirmed that job crafting is positively associated with workplace belongingness in Saudi Arabia. Al Wali et al. [52] affirmed that the dynamic capabilities of healthcare promote innovative work behaviour and job performance. However, job crafting has been well examined at the individual and firm levels. However, there is a scarcity of evidence on how job creation might improve personal and organisational results. Furthermore, research into the relationship between job crafting, patient care quality and nursing creativity is lacking. Thus, the following hypotheses have been formulated.

  H2a: TC has a positive relationship with patient QOC.
  H2b: TC has a positive relationship with employee creativity.
  H3a: RC has a positive relationship with patient QOC.
  H3b: RC has a positive relationship with employee creativity.
  H4a: CC has a positive relationship with patient QOC.
  H4b: CC has a positive relationship with employee creativity.

### 2.5. Patient QOC and Employee Creativity

**Figure 1:**
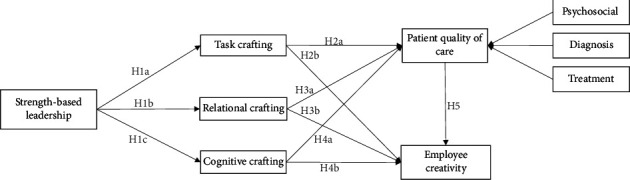
Research framework.

In the workplace, creativity is described as producing novel or distinctive concepts and approaches that are beneficial and useful [33, 53, 54]. The purpose of creative performance is not only to generate many ideas but also to develop those that effectively handle existing problems, create innovative products and services, thrive on market possibilities and improve organisational effectiveness. Ma et al. [55] argued that nurses' self-directed behaviour and teamwork positively promote creativity. The review work of Shahsavari Isfahani et al. [56] claimed that there is a positive synergy between patients' QOC and nurses' creativity. [57, 58] contended that nursing leaders and psychological safety help nurses promote creativity. Toyama and Mauno [59] argued that social support and emotional intelligence help to enhance nursing creativity in Japan. Finally, Slåtten et al. [60] affirmed that service QOC and leadership behaviour help to promote creativity among healthcare professionals in Norway. However, the concept of creativity has been well-grounded in nursing studies. Nevertheless, there is limited research on the relationship between patient QOC and creativity in the nursing profession. Thus, the following hypothesis has been proposed. Additionally, Figure 1 shows the conceptual framework.

  H5: Patient QOC has a positive relationship with employee creativity.

## 3. Methods

### 3.1. Study Design

A cross-sectional descriptive study design was employed to achieve the study objectives.

### 3.2. Study Setting

Based on the past studies, the study setting was based on a tertiary-level hospital in Henan province, China [10, 11, 16].

### 3.3. Participants

Based on the study design (cross-sectional), the endogenous constructs were continuous, and an analysis was performed through structural equation modelling (SEM). Therefore, the minimum sample size of nurses was calculated using G∗power software. Based on the software, the minimum sample size was 138. To account for a projected dropout rate of incomplete responses, an extra 30% of nurses were required [47, 61]. This approach is widely recommended in medical studies to sustain the statistical power and validity [62]. A two-phase sampling strategy was used to interact with the sample nurses. First, the number of nurses in each tertiary hospital was determined using stratified sampling. Second, convenience sampling was used to select nurses for each tertiary hospital. This study included licensed staff nurses who were employed during the study time-frame and had been working at their hospital for a minimum of six months. This study follows the guidelines of Luck et al. [63] and Weierbach et al. [64] to recruit potential nursing respondents. Luck et al. [63] suggested that the study's aim and possible outcomes must be aligned with nurses' concerns and context. Moreover, Weierbach et al. [64] affirmed that staff nurses should be involved in research decision-making. This strategy promotes community-based participatory action research by identifying stakeholders. Based on these, 675 nurses from Henan tertiary hospitals were invited to complete the online questionnaire, and 207 responses were received, resulting in a 30.60% effective rate.

### 3.4. Ethical Consideration

The Faculty of Nursing Ethics Committee gave ethical approval (No. 2024016). Throughout the research procedure, important ethical considerations such as participant well-being, privacy and anonymity were strictly followed. Participants were given detailed information about the study and informed of their ability to withdraw during the investigation. Access to sensitive participant data was limited to the authors, and all material was securely held in a locked cabinet. Furthermore, attendees were encouraged to ask any pertinent questions they had.

### 3.5. Measures

This research initially developed the items related to SBL, job crafting, patients' QOC and employee creativity in English. Based on the study by Lee et al. [65], an advanced version of the Brislin [66] model translated the Chinese version of these scales. A multiple-step translation process (Steps 1–9) was adopted to produce the final version of the questionnaire. All scale items were measured on a seven-point Likert scale.

#### 3.5.1. SBL

This study adapted the SBL scale from Wang et al.'s study [67]. This scale consists of two dimensions: the leader's SBL and the follower's SBL. The former alludes to the degree to which leaders recognise, cultivate and apply their abilities in the workplace. The latter pertains to how much a leader encourages followers to identify, cultivate and utilise their abilities [67].

#### 3.5.2. Job Crafting

In the current study, the job crafting scale was adapted from Saleh et al. [47], which was evaluated using 13 items of three dimensions. Five items measure the TC dimension. Four items measure the RC dimension. Finally, four items measure the CC.

#### 3.5.3. Patient's QOC

In this research, the items related to the patient's QOC were adapted from Li et al.'s study [17]. The QOC scale consists of three dimensions: psychological care (PC), diagnosis (DC), and treatment care (TOC). Four items were adapted to measure the PC. Five items were adapted to measure the DC; one item was excluded due to low factor loading. Finally, four items were adapted to measure the TOC.

#### 3.5.4. Employee Creativity

*Finally, the questionnaire related to employee creativity was adapted from past studies. A three-item scale was adapted from* Oldham and Cummings [68] and Malik et al. [53]. Finally, all the survey items/questionnaires are provided in Appendix A (see Table A1).

### 3.6. Data Collection and Analysis

The collected data were analysed through SPSS and SmartPLS. Before actual data analysis, pretesting and pilot testing steps were performed. Furthermore, the statistical assumptions of data analysis (normality, multicollinearity and outliers) were examined. The demographic, common method variance and descriptive analyses were performed through SPSS. The test of path analysis was performed through SEM using SmartPLS software [69]. Hair et al. [69] argued that PLS-SEM provides a comprehensive and consistent explanation of actual phenomena. Moreover, Hair et al. [69] contended that PLS-SEM is a superior technique to regression analysis. As Hair et al. [70] suggested that the data analysis in SEM consists of measurement and structural modelling. In measurement analysis, construct reliability and validity (convergent and discriminant) were performed, whereas in structural analysis, hypothesis testing was performed.

## 4. Results

### 4.1. Demographic and Common Method Variance

Table 2 displays participant information such as gender, age, education, experiences and position. The gender figures show a predominantly female workforce (59.42% vs. 40.58% male). Furthermore, age distribution figures show that the most excellent age group is 26–35 years, accounting for 43%, while the smallest age group is 46 years and above (11.11%). In terms of education, most nursing staff have a bachelor's degree (44.93%), followed by those with a master's degree (31.88%) and only 23.19% have an associate's degree. Furthermore, most participants (39.61%) had 3–5 years of experience, with those over 8 years being the least represented (13.04%). Lastly, the data show that 42.03% of participants have senior nursing positions, 8.70% have supervisory or higher positions and over half (49.28%) have nursing positions.

Common method bias (CMB) is a significant threat to data integrity that arises when one data collection method is used solely. Podsakoff et al. [71] suggested that both statistical and procedural techniques are adopted to reduce the threat. Procedurally, the survey questionnaire contained unequivocal statements of the research objectives. All research constructs were statistically tested using Harman's single-factor test, which showed that only 23.15% of the variance could be ascribed to a single factor, below the 50% threshold limit [71]. Furthermore, according to Kock [72] and Kock and Lynn [73], CMB is not a significant issue if all item variance inflation factors (VIFs) in SEM are less than 3.3.

### 4.2. SEM

#### 4.2.1. Measurement Analysis

As suggested by Hair et al. [69], measurement analysis was performed. In measurement analysis, reliability, convergent and discriminant validity analyses were performed. In terms of reliability, the composite reliability (CR) indicator was used with a threshold of ≥ 0.70 for acceptable reliability, and values between 0.60 and 0.70 were also acceptable [69]. To perform the convergent validity, average variance extracted (AVE) analysis was performed with a threshold of ≥ 0.50 [69, 70]. Moreover, indicator loading analysis was performed with a threshold of ≥ 0.708 excellent and 0.4–0.7 acceptable [69].  Table 3 shows the reliability and convergent validity analyses.

In the PLS-SEM literature, discriminant validity is examined using approaches such as the Fornell–Larcker criterion, cross-loadings and the heterotrait–monotrait correlation ratio (HTMT) [69]. However, Henseler et al. [74] challenged the Fornell–Larcker and cross-loading approaches for their inability in SEM. Hair et al. [70] used the HTMT criterion to assess discriminant validity, with a threshold value of ≤ 0.90.  Table 4 displays HTMT values for all study variables. The findings show that all constructs meet the minimum threshold of ≤ 0.90.

#### 4.2.2. Structural Analysis

To analyse the proposed hypotheses, Henseler et al. [74] suggested the bootstrapping technique with 5000 resamples. Moreover, Hahn and Ang [75] affirmed that the acceptance/rejection of hypotheses should be based on the confidence interval (CI). Thus, CI and *p* value (0.05) were adopted for the hypothesis's acceptance or rejection.

**Figure 2:**
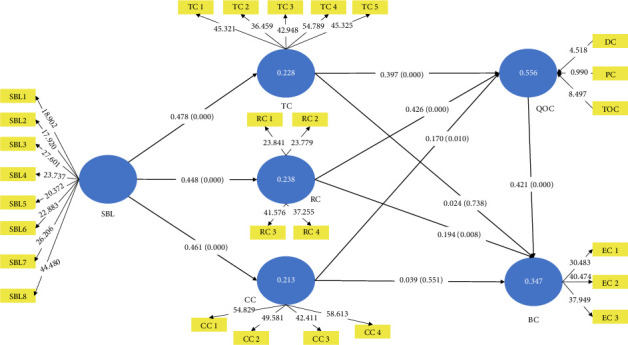
SEM path model.

Table 5 highlights the association between exogenous and endogenous variables. The analysis indicates that the relationship between SBL ⟶ TC (H1a: *β* = 0.478, *t*-value = 7.862), SBL ⟶ RC (H1b: *β* = 0.488, *t*-value = 7.623) and SBL ⟶ CC (H1: *β* = 0.461, *t*-value = 7.449) are positive and significant. Hence, H1a-c are accepted. Furthermore, the analysis highlights that the relationship between TC ⟶ QOC (H2a: *β* = 0.397, *t*-value = 5.888) is positive and significant. Therefore, H2a is accepted. Surprisingly, the analysis indicates that the relationship between TC ⟶ EC (H2b: *β* = 0.024, *t*-value = 0.334) is not statistically significant. Therefore, H2b is not supported. Moreover, the analysis shows that the relationship between RC ⟶ QOC (H3a: *β* = 0.426, *t*-value = 6.524) and RC ⟶ QOC (H3b: *β* = 0.194, *t*-value = 2.637) is positive and significant. Thus, H3a-b are supported. Additionally, the analysis depicted that the association of CC ⟶ QOC (H4a: *β* = 0.170, *t*-value = 2.571) is positive and significant. Thus, H4a is supported. On the other hand, the relationship of CC ⟶ EC (H4b: *β* = 0.039, *t*-value = 0.597) is insignificant. Therefore, H4b is rejected. Finally, the relationship between QOC ⟶ EC (H5: *β* = 0.421, *t*-value = 4.988) is positive and significant. Thus, H5 is supported. All the construct relationships are depicted in Figure 2. Additionally, Hair et al. [69] and Henseler et al. [74] suggested that the researcher does not necessarily need to examine the goodness-of-fit. Because the application of goodness-of-fit measures is still intermittent [74] and not fully developed in PLS-SEM. Therefore, there is no reason to evaluate and report it.

## 5. Discussion

In S5.0, to enhance the patient quality-of-care and nursing creativity, it is critical to comprehend how SBL, job crafting or AI crafting dimensions such as task, relational and cognitive interact. This knowledge will help build focused care and nursing outcomes. Thus, the purpose of this study is to investigate the impact of SBL in fostering AI-driven job crafting to improve patient QOC and nurse creativity. Based on job-demand resources' logic, the research framework is developed and analysed through the SEM technique.

The empirical results indicate that SBL has a positive and significant relationship with AI-driven job crafting variables (TC, RC and CC). The study findings are in line with Wang et al. [13], Wu et al. [51] and Saleh et al. [47]. Thus, H1a–c are supported. Park and Park [45] argued that leadership played an important role in providing autonomy and support. Wang et al. [13] claimed that SBL promotes nurses to use their best characteristics at work, leading to improved feelings of constructive impacts. This conclusion is noteworthy mainly because it emphasises leadership's crucial and primary role in boosting both patient and nursing outcomes. Unfortunately, the findings differ from other studies by Lavoie-Tremblay et al. [15]. Lavoie-Tremblay et al. [15] argued that SBL in nursing requires significant resources in a training program of 6-month spans. This substantial time commitment can strain already limited healthcare resources.

The results of the present study indicated that AI-driven job crafting dimensions (TC, RC and CC) have positively promoted patients' QOC and nursing creativity. Surprisingly, the relationship between TC and CC with nursing creativity was insignificant. Therefore, H2b and 4b were not supported. A possible justification for this is that simply altering the task or reframing one's mindset may not be sufficient to foster creativity. Creativity in nursing often depends on workplace challenges and pressures [76]. All of these external factors can stimulate creative problem-solving in nursing [77]. Therefore, the insignificant hypotheses (H2b and 4b) have logically justified that nurses tend to optimise the existing processes (patient care) rather than drive direct nursing creativity.

In terms of significant outcomes, the findings highlight that job crafting dimensions positively promote patient QOC. The present findings are supported by past studies [28, 44, 50]. These interactions may be justified by the fact that nurses can provide more individualised care by tailoring their tasks to the individual needs of patients [78]. Additionally, nurses can better handle complex patient demands when they communicate effectively with their colleagues [56]. Additionally, nurses might discover more meaning in their profession when they reframe how they think about patient care. Better patient care may result from the increased sense of purpose [13, 58, 78]. On the other hand, TC and CC have an insignificant association with nursing creativity. The current study findings are in line with past studies [79]. These results could be justified by the fact that TC entails changing the current tasks and can result in workflows that are more efficient. As opposed to investigating new ideas, nurses can find themselves more interested in streamlining current procedures. Similarly, while changing one's perspective on work can improve patient care, it may not always result in innovative ideas. Finally, the findings indicate that patients' QOC has a positive impact on nurses' creativity. The study findings are in line with the past studies [45, 47, 51].

Practically, AI-driven job crafting or AI crafting enhances the nursing workflow, decision-making and patient outcomes. For instance, Rosa et al. [80] developed an AI-based nursing workload classifier that achieved 72% accuracy. This AI algorithm allows nurses to manage their time effectively and gives more focus on patient-centred care. Likewise, Cho et al. [81] developed an AI-based “Nurse Healing Space” app to reduce nursing stress and job burnout. On the other hand, overreliance on AI in nursing management raises several ethical and safety challenges, such as data privacy and security [32, 82, 83]. Hassan and El-Ashry [84] argued that nurses may become overly reliant on AI and can risk their decision-making and clinical judgement skills.

## 6. Implications for Nursing Management

The present research has manifold implications for nursing management. Hospitals should place a high priority on developing management and leadership behaviours that support strength-based therapies. This can be accomplished through comprehensive training programs designed to improve leaders' capacity to discover and foster their nursing teams' specific strengths. Furthermore, it is critical to provide nurses with the tools and information they need to recognise and value SBL in their supervisors. By focussing on SBL, nursing leaders and staff are more engaged and motivated. This strategy is in complete alignment with the tenets of S5.0, which promotes a human-centred approach to healthcare technology integration. Moreover, hospitals can train leaders to adopt SBL effectively through real-world case studies, interactive modules and ongoing mentorship. It will help nursing leaders to foster practical application and sustained behavioural change. Secondly, the AI-driven job crafting gives nurses the ability to personalise their responsibilities in ways that greatly increase their creativity and patient care. This proactive approach fosters a deep sense of freedom and meaning in the nursing profession by allowing them to modify their tasks and improve their relationships with patients. In the rapidly evolving healthcare industry, where technological integration is essential, this kind of flexibility is crucial. By empowering nurses to take care of their duties, AI-driven job crafting not only promotes creativity but also fosters inventive solutions to satisfy patient needs effectively. Moreover, hospital management can implement AI-driven job crafting by involving nursing staff in codesigning AI tools. Consequently, it will enhance autonomy and routine tasks and align with core nursing job design (patient care). Finally, SBL and AI-driven job crafting together can help nursing teams develop an innovative culture. Healthcare organisations can improve their ability to effectively adapt to challenges by fostering an environment that supports and encourages creativity. As nurses use their creativity to create novel solutions for patients' needs, this innovative culture not only helps nurses by giving them chances for professional development but also results in better patient care.

## 7. Limitations and Future Directions

Apart from contributions, this study has several limitations that pave the way for future research. First and foremost, the study employs a cross-sectional survey method, which restricts the capacity to determine causal limitations. Moreover, it restricts the generalisability of the outcomes. To overcome this, future research may perform a longitudinal study to overcome these limitations. Secondly, the study relies on self-reported data, which introduce the social desirability bias. Thus, future research may adopt mixed-methods approach to reduce this bias. Finally, the data were collected from one country (China), which may limit the generalisability and sampling bias. Therefore, future research may be performed in other geographic areas to promote the external validity of the findings.

## Figures and Tables

**Table 1:** Past studies on AI and job crafting.

Authors	Focus	Sample descriptions	Outcomes	Limitations
Cheng et al. [19]	Impact of AI adoption on employee promotion- and prevention-focused job crafting.	Employees working in Chengdu China.	Organisational AI adoption promotes the promotion-focused job crafting.	Perform in other organisational setting.

Dong et al. [18]	To propose that AI promotes AI self-efficacy which enhance the proactive coping behaviour to adapt to AI or AI crafting.	Employees working in China	AI crafting is an emerging dimension to comprehensively understand the AI in the workplace.	Negative effect of AI on performance.

Gerçek et al. [33]	To examine the mediating role of job crafting between resilience and career satisfaction and moderating role of smart technology, AI, robotics and algorithms (STARA) awareness on resilience and job crafting relationship.	White collar employees working in Turkey firms	High level of STARA awareness negatively moderates resilience and job crafting relationship. Low level of STARA awareness positively affects the resilience and job crafting association.	To understand how employee perceive the implementation of STARA to make their attitudes about these technologies.

Handke et al. [21]	To explore the use of information and communication technologies to redesign their job (job crafting).	Interview with German University employees	All ICT resources positively promote job crafting dimensions.	Apply ICT for job crafting in a variety of work setting. Investigate the new ICT use crafting against the background of AI in the workplace.

He et al. [34]	To examine the role of leader AI symbolisation to promote employee job crafting behaviours among USA and Chinese employees.	Experiment and multiwave survey from employees working in USA and China.	Leaders' AI symbolisation promotes employee change readiness and job crafting.	Application of AI in diverse industrial setting such as healthcare, manufacturing and finance. Dark side of AI

He et al. [35]	To examine the role of AI awareness to promote service performance through job crafting (as motivation process) and moderating role of AI knowledge.	Employee working in hospitality sector in China.	AI knowledge (challenge appraisal) positively effect on job crafting and service performance, and this relationship is moderator by AI knowledge.	Examine the impact of AI in other industries/sectors.

Li et al. [17]	To investigate the leader AI crafting and employee AI crafting to enhance employee AI engagement and help.	Employees working in restaurants in China	Leader and employee AI crafting positively promotes employee AI engagement and help.	Work on other types of AI crafting at leader and employee levels. Nature of task an important boundary conditions in studying employee AI crafting.

Perez et al. [22]	To qualitative investigate that how radiologists perceive AI and respond through job crafting and identity work	Radiologists working in French healthcare systems.	From AI perspective, three categories of job crafting emerge; approach, avoidance and identity crafting.	Other areas in healthcare. Focus on other outcomes such as patient's outcomes and satisfaction.

Song et al. [23]	To investigate the employee perceptions on job crafting towards employee-robot collaboration in hospitality.	Employee working in hotel chain in China	Employee perceptions have positive association with job crafting.	Consider the bottom-up proactive work behaviour such as role characteristics (central vs. peripheral) and technology readiness (high vs. low).

Perez et al. [36]	To examine that how learning algorithms affected employee working conditions, autonomy and meaning of their work (job crafting).	Employee working in French banking sector.	AI empowered job crafting successfully alter employee experience of work and enhance the human value of their services.	Apply in other industrial setting. Apply from employee perspective across three job crafting dimensions.

Tan et al. [37]	To examine the effect of workplace anxiety of AI on job crafting and competitive productivity.	Employee working in hospitality sector in China.	Workplace anxiety of AI promotes job crafting through conservation of resources.	Applied in other industries. Gender, age, education and experiences affect the outcomes.

Teng et al. [38]	To examine the role of STARA awareness on work engagement and proactive customer service performance	Employee working in hotel sector in USA	STARA awareness has direct and negative effect on proactive customer services awareness.	Applied in other sectors.

Tong et al. [39]	To explore the role of Gen AI to help nurses to strengthen their research skills.	Nurses working in Chinese hospital.	Gen AI enhances nurses research skills such as utilisation efficacy, booster research, role reversal and beautiful dream.	Applied in other countries and industries.

Zhao et al. [40]	To examine that how AI dependence influences employee job crafting among hotel employees	Hotel employee working in China	Employee perceived AI dependence as a positive stimulus, resulting in higher job crafting.	Performed in cross-culture and cross-country aspects.

**Table 2:** Demographic analysis.

Variable	*N*	%
Gender		
Female	123	59.42
Male	84	40.58
Age		
≤ 25	52	25.12
26–35	89	43.00
36–45	43	20.77
≥ 46	23	11.11
Education		
Associate degree or below	48	23.19
Bachelor degree	93	44.93
Master degree	66	31.88
Years of services		
≤ 2 years	51	24.64
3–5 years	82	39.61
6–7 years	47	22.71
≥ 8 years	27	13.04
Position		
Nurses	102	49.28
Senior nurses	87	42.03
Supervisor nurse or above	18	8.70

**Table 3:** Reliability and convergent validity.

Dimensions	Items	Loadings	VIF	CR	AVE
Strength-based leadership (SBL)	SBL1	0.691	1.610	0.889	0.557
	SBL2	0.694	1.600		
	SBL3	0.767	1.891		
	SBL4	0.736	1.721		
	SBL5	0.715	1.705		
	SBL6	0.737	1.742		
	SBL7	0.774	1.937		
	SBL8	0.841	2.501		

Task crafting (TC)	TC1	0.858	2.591	0.916	0.741
	TC2	0.847	2.512		
	TC3	0.856	2.429		
	TC4	0.877	2.761		
	TC5	0.867	2.602		

Relational crafting (RC)	RC1	0.762	1.587	0.841	0.649
	RC2	0.769	1.665		
	RC3	0.856	1.915		
	RC4	0.832	1.749		

Cognitive crafting (CC)	CC1	0.877	2.484	0.899	0.757
	CC2	0.857	2.219		
	CC3	0.857	2.396		
	CC4	0.889	2.562		

Psychosocial care (PC)	QOC1	0.840	1.996	0.867	0.713
	QOC2	0.847	2.134		
	QOC3	0.857	2.136		
	QOC4	0.834	1.984		

Diagnosis care (DC)	QOC5	0.839	1.839	0.848	0.681
	QOC6	0.825	1.865		
	QOC7	0.803	1.779		
	QOC8	0.833	1.996		

Treatment care (TOC)	QOC10	0.895	3.106	0.94	0.838
	QOC11	0.926	2.222		
	QOC12	0.931	3.288		
	QOC13	0.911	3.210		

Employee creativity (EC)	EC1	0.823	1.657	0.798	0.707
	EC2	0.847	1.717		
	EC3	0.852	1.663		

**Table 4:** Discriminant validity.

Variables	SBL	TC	RC	CC	PC	DC	TOC	EC
SBL								
TC	0.525							
RC	0.566	0.303						
CC	0.515	0.423	0.357					
PC	0.612	0.423	0.352	0.393				
DC	0.465	0.587	0.403	0.383	0.462			
TOC	0.695	0.511	0.692	0.453	0.497	0.513		
EC	0.522	0.389	0.559	0.353	0.302	0.555	0.596	

**Table 5:** Hypotheses analysis.

Relation	*β*-value	*t*-value	C.I [2.05%–97.5%]	*p* value	Decision
H1a: SBL ⟶ TC	0.478	7.862	[0.346–0.587]	0.001	Supported
H1b: SBL ⟶ RC	0.488	7.623	[0.352–0.603]	0.001	Supported
H1c: SBL ⟶ CC	0.461	7.449	[0.329–0.573]	0.001	Supported
H2a: TC ⟶ QOC	0.397	5.888	[0.270–0.536]	0.001	Supported
H2b: TC ⟶ EC	0.024	0.334	[−0.102–0.174]	0.738	Not supported
H3a: RC ⟶ QOC	0.426	6.524	[0.298–0.553]	0.001	Supported
H3b: RC ⟶ EC	0.194	2.637	[0.048–0.334]	0.008	Supported
H4a: CC ⟶ QOC	0.170	2.571	[0.039–0.299]	0.01	Supported
H4b: CC ⟶ EC	0.039	0.597	[−0.088–0.169]	0.551	Not supported
H5: QOC ⟶ EC	0.421	4.988	[0.241–0.575]	0.001	Supported

**Table 6:** Survey Questionnaire.

Items	References
*Strength-based leadership*	
SBL1: My leader knows when I am at my best, including in using AI tools in patient care.	Wang et al. [67]
SBL2: My leader focuses on what I am good at, including my ability to use AI in clinical tasks.	
SBL3: My leader appreciates my strengths, including those related to AI-supported care.	
SBL4: My leader helps me to discover my strengths, including how I can effectively use AI in my role.	
SBL5: My leader helps me to do my job in a manner that best suits my strong points, including the use of AI tools.	
SBL6: My leader gives me the opportunity to do what I am good at, including AI-assisted nursing tasks.	
SBL7: My leader ensures that my job tasks, including those involving AI, are aligned with my strengths.	
SBL8: My leader discusses with me how I can further my strengths, including skills in using AI technologies.	

*AI job crafting or AI crafting*	
Task crafting: In our ward	Saleh et al. [47]
TC1: The proficiency levels of newcomers in using AI tools are properly understood to help them grow.	
TC2: Young team members are given AI-supported tasks which provide them with opportunities to grow.	
TC3: Each team member is preferentially allocated to AI-enhanced tasks which provide them with opportunities to grow.	
TC4: AI-based tools (e.g., clinical decision support systems, predictive analytics and digital checklists) to provide better care for patients are regularly introduced for team members to utilise.	
TC5: Team members collaboratively come up with the AI-assisted workflow for each work shift to provide the most suitable care for patients.	
Relational crafting: In our ward	
RC1: Positive events involving the use of AI tools are shared among team members to reaffirm the value of our work.	
RC2: The work accomplished when caring for a patient, including AI-assisted decisions, is shared among team members to reassess the meaning of care in our work.	
RC3: The capabilities of each team member, including their skills in using AI tools, are being fully utilised to provide better care for the patient as a team.	
RC4: Work decisions, especially those involving AI-supported care, are made with consent from all team members to improve satisfaction.	
Cognitive crafting: In our ward	
CC1: A patient's information, including AI-generated insights, is shared among team members even outside of meetings to provide better care for the patient as a team.	
CC2: The workplace atmosphere allows team members to easily and voluntarily confide in others about their worries or problems, including those related to using AI tools.	
CC3: Topics aside from work, including experiences with AI in care settings, are brought up in everyday conversations to improve relationships between team members.	
CC4: The process of sharing AI-assisted patient care methods within the team has been established to provide consistent care between nurses.	

*Quality of care (psychosocial care, diagnosis and treatment care)*	
QOC1: I ensure patient privacy is protected during care, including when using digital health records.	Li et al. [17]
QOC2: I offer emotional support to patients, utilising digital communication tools	
QOC3: I communicate effectively with patients and their families, sometimes using tele-health platforms	
QOC4: I respect patients' dignity, both in person and when interacting via digital	
QOC5: I respond promptly to patients' needs, aided by digital monitoring	
QOC6: I strictly implement the doctor's orders, including those delivered through electronic health systems	
QOC7: I accurately perform nursing operations, sometimes using digital devices	
QOC8: I closely monitor patients' conditions, utilising digital monitoring devices	
QOC9: I provide health education to patients, occasionally using digital platforms	
QOC10: I ensure medication safety for patients, with the help of electronic prescribing systems	
QOC11: I actively participate in nursing quality management activities, including those supported by digital dashboards	
QOC12: I strictly implement infection control measures, sometimes guided by digital protocols	
QOC13: I carefully check and verify the identity of patients, using digital ID systems	

*Employee creativity*	
EC1: The nurse's work is original and practical, utilising digital technologies to develop ideas, methods or products that are both unique and especially useful to the organisation.	Malik et al. [53] and Oldham and cummings [68]
EC2: The nurse's work is adaptive and practical, applying existing digital tools to develop ideas, methods or products that are useful to the organisation.	
EC3: The nurse's work is creative, demonstrating the ability to develop ideas, methods or products that are both original and useful to the organisation, sometimes through the innovative use of digitalisation.	

## Data Availability

The data that support the findings of this study are available on request from the corresponding author. The data are not publicly available due to privacy or ethical restrictions.
